# SRSF3 maintains transcriptome integrity in oocytes by regulation of alternative splicing and transposable elements

**DOI:** 10.1038/s41421-018-0032-3

**Published:** 2018-06-19

**Authors:** Dang Vinh Do, Bernhard Strauss, Engin Cukuroglu, Iain Macaulay, Keng Boon Wee, Tim Xiaoming Hu, Ruiz De Los Mozos Igor, Caroline Lee, Andrew Harrison, Richard Butler, Sabine Dietmann, Ule Jernej, John Marioni, Christopher W. J. Smith, Jonathan Göke, M. Azim Surani

**Affiliations:** 1 Wellcome Trust/Cancer Research UK Gurdon Institute, University of Cambridge, Tennis Court Road, Cambridge, CB2 1QN UK; 2 Department of Physiology, Development and Neuroscience, University of Cambridge, Downing Street, Cambridge, CB2 3DY UK; 3 Computational and Systems Biology, Genome Institute of Singapore, 60 Biopolis Street, Singapore, 138672 Singapore; 4 Earlham Institute, Norwich Research Park, Norwich, NR4 7UH UK; 5 Department Fluid Dynamics, Institute of High Performance Computing, 1 Fusionopolis Way, Singapore, 138632 Singapore; 6 Biomolecular Function Discovery Division, Bioinformatics Institute, 30 Biopolis Street, Singapore, 138671 Singapore; 7 EMBL European Bioinformatics Institute, Wellcome Genome Campus, Hinxton, CB10 1SD, Cambridge, UK; 8 Department of Molecular Neuroscience, UCL Institute of Neurology, London, UK; 9 Wellcome Trust Medical Research Council Stem Cell Institute, University of Cambridge, Tennis Court Road, Cambridge, CB2 1QR UK; 10 Cancer Research UK Cambridge Institute, University of Cambridge, Li Ka Shing Centre, Robinson Way, Cambridge, CB2 0RE UK; 11 Wellcome Trust Sanger Institute, Wellcome Genome Campus, Hinxton, Cambridge, CB10 1SA UK; 12 Department of Biochemistry, University of Cambridge, Tennis Court Road, Cambridge, CB2 1QW UK

## Abstract

The RNA-binding protein SRSF3 (also known as SRp20) has critical roles in the regulation of pre-mRNA splicing. Zygotic knockout of *Srsf3* results in embryo arrest at the blastocyst stage. However, SRSF3 is also present in oocytes, suggesting that it might be critical as a maternally inherited factor. Here we identify SRSF3 as an essential regulator of alternative splicing and of transposable elements to maintain transcriptome integrity in mouse oocyte. Using 3D time-lapse confocal live imaging, we show that conditional deletion of *Srsf3* in fully grown germinal vesicle oocytes substantially compromises the capacity of germinal vesicle breakdown (GVBD), and consequently entry into meiosis. By combining single cell RNA-seq, and oocyte micromanipulation with steric blocking antisense oligonucleotides and RNAse-H inducing gapmers, we found that the GVBD defect in mutant oocytes is due to both aberrant alternative splicing and derepression of B2 SINE transposable elements. Together, our study highlights how control of transcriptional identity of the maternal transcriptome by the RNA-binding protein SRSF3 is essential to the development of fertilized-competent oocytes.

## Introduction

Development of fertilization-competent oocytes includes completion of meiosis, cytoplasmic maturational events that provide competence for fertilization and embryogenesis, and maintenance of genomic integrity by protection against disruptive factors such as retrotransposon activation^1^. These important processes are largely dependent on mRNA and proteins that are synthesized and stored in oocytes as maternally inherited factors during their growth phase^2, 3^. Growing oocytes with an intact germinal vesicle (GV) are arrested at prophase I (referred to as fully grown GV oocytes) at the end of their growth phase. Following their induction by luteinizing hormone, fully grown GV oocytes undergo germinal vesicle breakdown (GVBD) and resume meiosis. Meiosis I commences with the assembly of the meiotic spindle and is completed when the oocytes extrudes the first polar body. Meiotic maturation is complete when the oocytes are arrested at metaphase of meiosis II (referred as MII oocytes)^2^. Because there is a transition from the transcriptionally active state in growing GV oocytes, to a transcriptionally inactive state in the fully grown GV and MII oocytes^4^, it is necessary to generate a sufficient pool of maternal transcripts, while maintaining the transcriptome integrity in the oocyte.

One of the most important contributors to transcriptome complexity is pre-mRNA alternative splicing (AS)^5, 6^. The vast majority (89% ensemble Version 82) of protein-coding genes in the mouse genome undergo AS. The correct combination of exons through AS ensures that gene isoforms are expressed that are required for the specific context. AS can result in expression of alternative protein isoforms that have distinct functions^7^, and defects in splicing control can result in loss-of-function with severe phenotypes observed in pluripotent cells, development, and disease models^8, 9^. The presence of conserved stage-specific transcript variants in mouse and human MII oocytes^10–13^ suggests that control of splicing plays a central role in regulation and establishment of the maternal transcriptome. However, the factors that contribute to the regulation of AS and transcriptome integrity in oocytes are still largely unknown.

Serine*/*arginine-rich splicing factor 3 (SRSF3 or SRp20) is an RNA-binding protein belonging to a highly conserved family of serine/arginine-rich (SR) proteins^14^. Like other members of the SR protein family, SRSF3 is best known as a splicing factor and regulator of AS^15–20^, but it also participates in many other posttranscriptional processes, including RNA polyadenylation^21^, RNA export^22^, pri-miRNA processing^23^, and internal ribosome entry site-mediated translation of a viral RNA^24^. SRSF3 is essential for preimplantation embryo development^25^, however, its contribution to the maternal transcriptome has not been reported. Here we find that loss of SRSF3 function in mouse oocytes severely impairs the maternal transcriptome, substantially compromising the capacity of entry into meiosis. By analyzing single cell RNA-Seq data from SSRF3 mutant fully grown GV oocytes, we identify pervasive splicing aberrations that partially explain the observed phenotype. Surprisingly, SRSF3 depletion also induces a dramatic shift in transcriptome composition characterized by increased expression of B2 short interspersed nuclear element (SINE) retrotransposons in mutant oocytes. Together our study highlights how precise control of the maternal transcriptome by RNA-binding proteins is important for the growth and development of fertilized oocytes.

## Results

### Depletion of maternal SRSF3 protein results in developmental arrest at one/two-cell stage

**Fig. 1:**
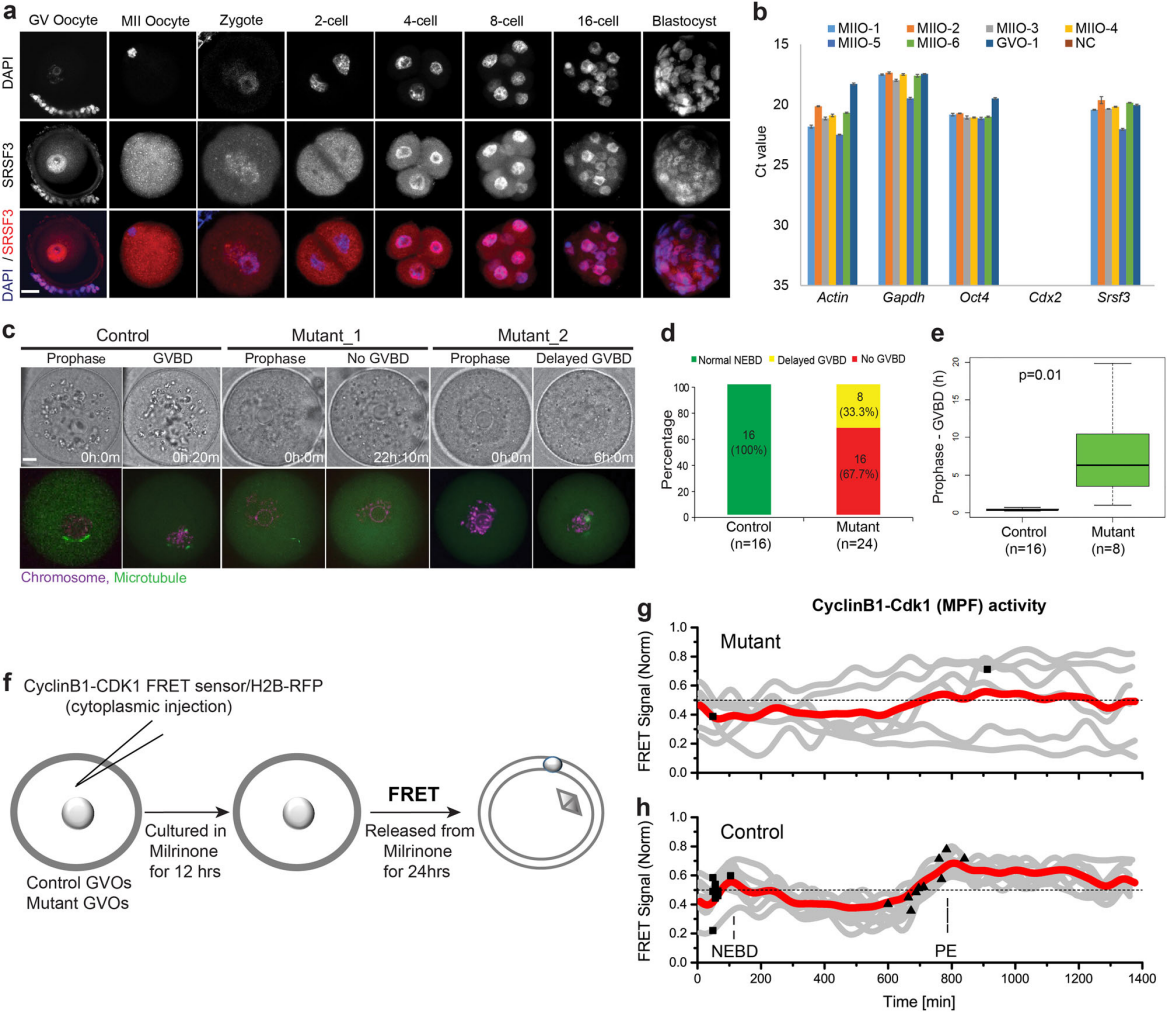
*Srsf3* knockout oocytes show a major defect in meiotic resumption. **a** Immunostaining shows SRSF3 protein expression in GV oocytes, MII oocytes and preimplantation embryos. Scale bar: 100 µm. **b** Single cell quantitative real-time PCR shows *Srsf3* mRNA expression in MII oocytes and GV oocytes. NC negative control, M2O MII oocyte, GVO GV oocyte. **c** Time-lapse confocal live imaging of control and mutant oocytes microinjected with H2B-RFP and EB3-mEGFP RNAs. Chromosome in magenta, microtubule in green. Scale bar: 20 µm, h hour, m minute, GVBD: nuclear envelope breakdown. **d** A graph shows the percentage of control and mutant oocytes undergoing normal GVBD, delayed GVBD, or no GVBD. Numbers of oocytes examined are shown under the graph. **e** A box plot shows timing from prophase I to GVBD in control and mutant oocytes. Numbers of oocytes examined are shown under the graph. *p*-value was calculated by two-tailed Student’s *t*-test. **f** Schematic illustration of Förster resonance energy transfer (FRET) experiment using a CDK1 FRET sensor to visualize CDK1 activation in control and mutant oocytes during meiosis. Milrinone was used to prevent oocytes from entering meiosis. **g** Gray lines are FRET curves of mutant oocytes. Red line is the mean of FRET curves of six mutant oocytes. Black squares represent the time point of GVBD. Only two mutant oocytes underwent GVBD during the course of live imaging. **h** Gray lines are FRET curves of control oocytes. Red line is the mean of FRET curves of ten control oocytes. Black squares represent time point of GVBD and black triangles represent the time point of the completion of polar body extrusion (PE), which is the first frame showing complete abscission of the polar body

Although, *Srsf3*-zygotic knockout embryos die before the blastocyst stage^25^, the role of oocyte-derived SRSF3 in the oocyte is unknown. We first assessed whether SRSF3 protein and mRNA are present in oocytes and preimplantation embryos using immunofluorescence (IF) and single cell quantitative PCR (Fig. 1a, b). Our results showed *Srsf3* was highly expressed in GV and MII oocytes (at metaphase of meiosis II), and it is therefore a maternal factor. Because SRSF3 is present in oocytes, conventional zygotic knockout (*Srsf3*^−/−^) embryos would have this maternally inherited protein during early embryogenesis. We therefore established maternal *Srsf3*-knockout oocytes (referred as *Zp3-Cre*^*+*^*, Srsf3*^*f/f*^) using a Zp3-Cre mating strategy, which leads to a loss of function exclusively in all female gametes (Supplementary Figure S1a)^26^. IF analysis confirmed the absence of SRSF3 in the mutant oocytes (Supplementary Figure S1b). Five maternal knockout females were mated with wild-type males, but none of them produced live progeny (data not shown).

To determine the possible causes of sterility, we examined the development of maternal knockout embryos collected from mutant *Zp3-Cre*^*+*^, *Srsf3*^*f/f*^ females mated with wild-type males (Supplementary Figure S1c). From five mated *Zp3-Cre*^*+*^, *Srsf3*^*f/f*^ females that had plug, we only obtained fertilized embryos from only one mice. In addition, during in vitro culture the mutant embryos were arrested at the one- or two-cell stage (Supplementary Figure S1d), and all of them lacked the SRSF3 protein as judged by IF (Supplementary Figure S1e). Taken together, these results indicate that maternal SRSF3 is essential for preimplantation development.

### *Srsf3*-knockout oocytes exhibit severe GVBD defects

To determine the cause for the early arrest of maternal knockout embryos following SRSF3 depletion in the oocyte, we performed 3D time-lapse confocal live imaging and visualized microtubules with EB3-eGFP and DNA with H2B-RFP during in vitro meiotic maturation. For control fully grown GV oocytes, we found that GVBD was followed by spindle assembly relocation to the oocyte surfaces, with segregation of one set of the chromosomes into the first polar body. A second spindle assembly followed, and the egg was arrested at the metaphase II stage (referred as MII oocytes) (Supplementary Movie S1, Fig. 1c). In mutant oocytes, we observed two mutually exclusive phenotypes. The first phenotype was characterized by a lack of GVBD (Supplementary Movie S2, Fig. 1d), which we detected in 66.7% (16 out of 24) of all mutant oocytes (Fig. 1e). The second phenotype seen in 33.3% of mutant oocytes (8/24), manifested in a delayed GVBD and a meiotic spindle that eventually collapsed (Supplementary Movie S3, Fig. 1c–e). Both phenotypes appeared to result from a defect in GVBD in mutant oocytes.

A major regulator of GVBD is the maturation promoting factor (MPF), a complex consisting of CDK1 (CDC2) and a regulatory subunit cyclin B1^27^. To investigate whether the GVBD defect observed in mutant oocytes is associated with dysregulation of MPF activity, we performed Förster resonance energy transfer (FRET) experiments using a FRET biosensor^28^ that detects cyclin B1-CDK1 kinase phosphorylation and thus kinase in control and mutant fully grown GV oocytes during in vitro maturation (Fig. 1f). In all control oocytes, we found a similar pattern of CyclinB1-CDK1 activity in which CDK1 was activated shortly before GVBD. Subsequently, CDK1 activity gradually decreased, and then peaked again at polar body extrusion (Fig. 1h). In contrast, mutant oocytes showed variably fluctuating CDK1 activity, and no major activation peak of CDK1 was observed before GVBD (Fig. 1g). These results suggested that the observed GVBD defect in mutant oocytes is possibly caused by an upstream disruption of meiotic entry and not by events involved in the disassembly of the nuclear membrane.

### SRSF3 knockout in oocytes results in numerous changes in AS

**Fig. 2:**
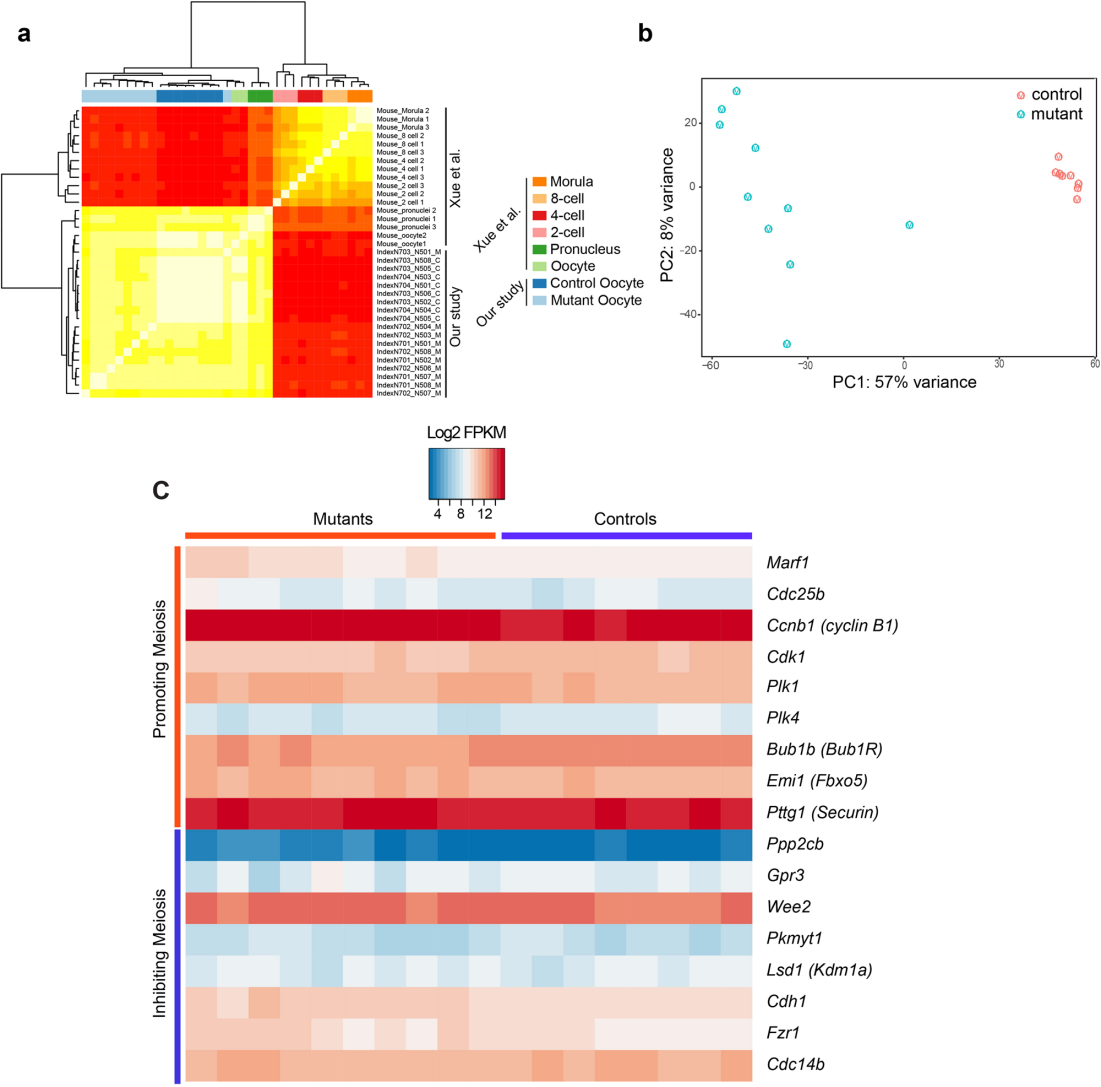
Aberrant transcriptome in *Srsf3* mutant oocytes. **a** A heatmap shows the Spearman correlation of gene expression between control and mutant oocytes in our single cell RNA-Seq data and oocytes and early mouse preimplantation embryos in previously published single cell RNA-seq data^29^. **b** PCA plot using the 5000 genes with the highest variance across all samples, axes are labeled to include the percentage of variance explained. **c** A heatmap showing the transcript level of key genes that promote or inhibit meiosis in control and mutant oocytes. The color indicates the expression level

To investigate the molecular mechanism of the GVBD defect, we performed single cell RNA-seq for control and mutant oocytes. Both control and mutant samples clustered together with publicly available RNA-Seq data from mouse oocytes, and separately from other preimplantation embryos^29^ (Fig. 2a). When we compared the mutant and control expression profiles, we found that they form two distinct groups (Fig. 2b). Interestingly, mutant oocytes showed higher variation in their gene expression profiles compared to control oocytes, possibly reflecting the higher variation in phenotypes of mutant oocytes compared to strict progression observed in control oocytes, even though no clear subpopulations are distinguishable (Fig. 2b). We found that 3190 genes (1440 downregulated genes and 1750 upregulated genes) showed significant (more than 2-fold) differences in transcript levels between mutant and control (Supplementary Table S2). Nevertheless, we found no significant change in transcript levels of most meiosis-related genes including the MPF genes *Cdk1*, *Ccnb* (cyclin B1)^27^, the promoting factors *Cdc25b*^30^, *Marf1*^1^, *Plk1*^31^, *Plk4*^32^, *Bub1b*^33^, *Emi1*^34^ and *Securin*^35^, or the inhibiting factors *Ppp2cb*, *Gpr3*^36^, Wee2^37^, *Pkmyt1*^38^, *Kdm1a*^39^, *Cdh1*, *Fzr1*^40, 41^, and *Cdc14b*^42^ (Fig. 2c).

**Fig. 3:**
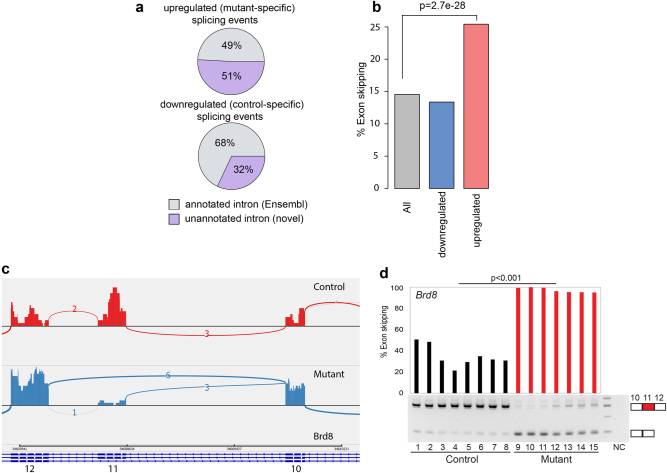
Aberrant alternative splicing in *Srsf3-*mutant oocytes. **a** Pie charts show percentages of annotated and unannotated splicing events in upregulated (mutant-specific) and downregulated (control-specific) splicing events. **b** A bar graph shows percentages of exon skipping in unannotated splicing events. All splicing events in gray, downregulated (control-specific) splicing events in blue, upregulated (mutant-specific) splicing events in red. **c** A representative sashimi plot shows an exon skipping event in *Brd8* transcript in control and mutant oocyte. The numbers of junction reads between two connecting exon are shown in the sashimi plot. **d** Validation of the exon skipping on *Brd8* transcript in control and mutant oocyte by semiquantitative PCR. Percentages of exon skipping calculated for eight control and seven mutant oocytes are shown on the top. Mann–Whitney test (Wilcoxon rank sum test) was used to calculate *p*-value

Since SRSF3 is an important regulator of AS^14^, we asked whether SRSF3 depletion results in an altered splicing pattern. To detect novel AS events, we counted all split reads (reads that map to two different parts in the genome, “candidate splicing events”). We then calculated the fold change of the read counts at every candidate splicing event^43^. As we are interested in a change of junction usage relative to all other junctions of the same gene, we calculated differential splicing for every gene separately, thereby correcting for the overall change in gene expression. Our results show an enrichment of unannotated splicing events in mutant oocytes compared to controls (51% in mutants vs. 32% in controls) (Fig. 3a), suggesting that AS in *Srsf3* mutant oocytes are strongly affected leading to a high number of isoforms that have not been described in any context. While some of these previously unannotated events are probably genuine regulated AS events, it is possible that a subset corresponds to “aberrant” events that do not occur at high frequency in any wild-type context. These events are likely to change the coding sequence of gene isoforms, thereby possibly changing the protein function, and in some cases leading to nonfunctional gene isoforms (Supplementary Table S3). This analysis also confirms an enrichment in exon skipping events in mutant oocytes (Fig. 3b). Together we find that SRSF3 is essential for maintaining splicing integrity, most commonly by promoting exon inclusion in wild-type mouse oocytes.

To directly address the relationship between SRSF3-regulated splicing activity and RNA binding, we analyzed published data of crosslinking and immunoprecipitation (CLIP) experiments in embryonic carcinoma cells^44^. An SRSF3-regulated splicing map^45^ was generated using the SRSF3 CLIP binding datasets on the SRSF3-regulated cassette exons in control and mutant oocytes. We found that SRSF3 binding is elevated within SRSF3-enhanced alternative exons that are also known as mutant-specific skipped exons (Supplementary Figure S2, red trace) compared to SRSF3-silenced exons (blue trace) or control-skipped exons not regulated by SRSF3 (gray trace). Binding was not elevated in the long intronic regions flanking enhanced exons (Supplementary Figure S2, red trace). There is also no obvious elevation of SRSF3 binding in the flanking constitutive exons, their immediate intron flanks and all regions associated with SRSF3-silenced alternative exons (Supplementary Figure S2, blue trace). These results suggest that SRSF3 binds directly to SRSF3-enhanced cassette exons and promotes inclusion of the exons in the mature transcript

### Misregulated *Brd8*- and *Pdlim7*-AS contributes to GVBD defect in *Srsf3*-knockout oocytes

As a starting point for investigating the contribution of individual misregulated AS events to the GVBD phenotype, we selectively validated a number of exon skipping events predicted to be regulated by SRSF3 in control and mutant oocytes, focusing on genes with functions associated with the GVBD phenotype: *Brd8* (Bromodomain 8), *Pdlim7* (PDZ and LIM domain 7), and *Npm2* (nucleoplasmin 2). BRD8 contains a Bromodomain, which binds acetylated lysines, and is involved in the regulation of histone acetyl transferase activity, chromatin remodeling, and transcription^46^. *Brd8-*knockdown colon cancer cells are particularly sensitive to microtubule spindle poisons^47^, suggesting its potential role in protecting microtubule spindle. PDLIM7 is an actin-associated protein that has the role in the assembly of an actin filament-associated complex^48^. Zygotic knockout of *Pdlim7* in mice results in postnatal lethality^48^. NPM2 is known as an oocyte-derived factor that is essential for nucleolar organization and early embryonic development^49^.

We performed RT-PCR on single control and mutant oocytes using primers flanking the skipped exons of these genes (Supplementary Table S1) and the percentage of exon skipping was determined. Our results confirmed increased skipping of exon 11 of *Brd8* (Fig. 3c, d), exons 2 and 3 of *Npm2* (Supplementary Figure S3a, S3b) and a mutually exclusive switch from use of *Pdlim7* exon 5 to exon 6 (Supplementary Figure S3c, S3d). The AS events of *Brd8* and *Pdlim7* are both annotated in Ensembl and maintain the same reading frame, whereas the exon skipping event of Npm2 is newly identified in this study and is predicted to lead to nonsense-mediated mRNA decay (NMD) by frameshifting. Taken together, these results suggest that SRSF3 has a critical role in promoting exon inclusion to ensure correct splicing and translation of functional proteins.

**Fig. 4:**
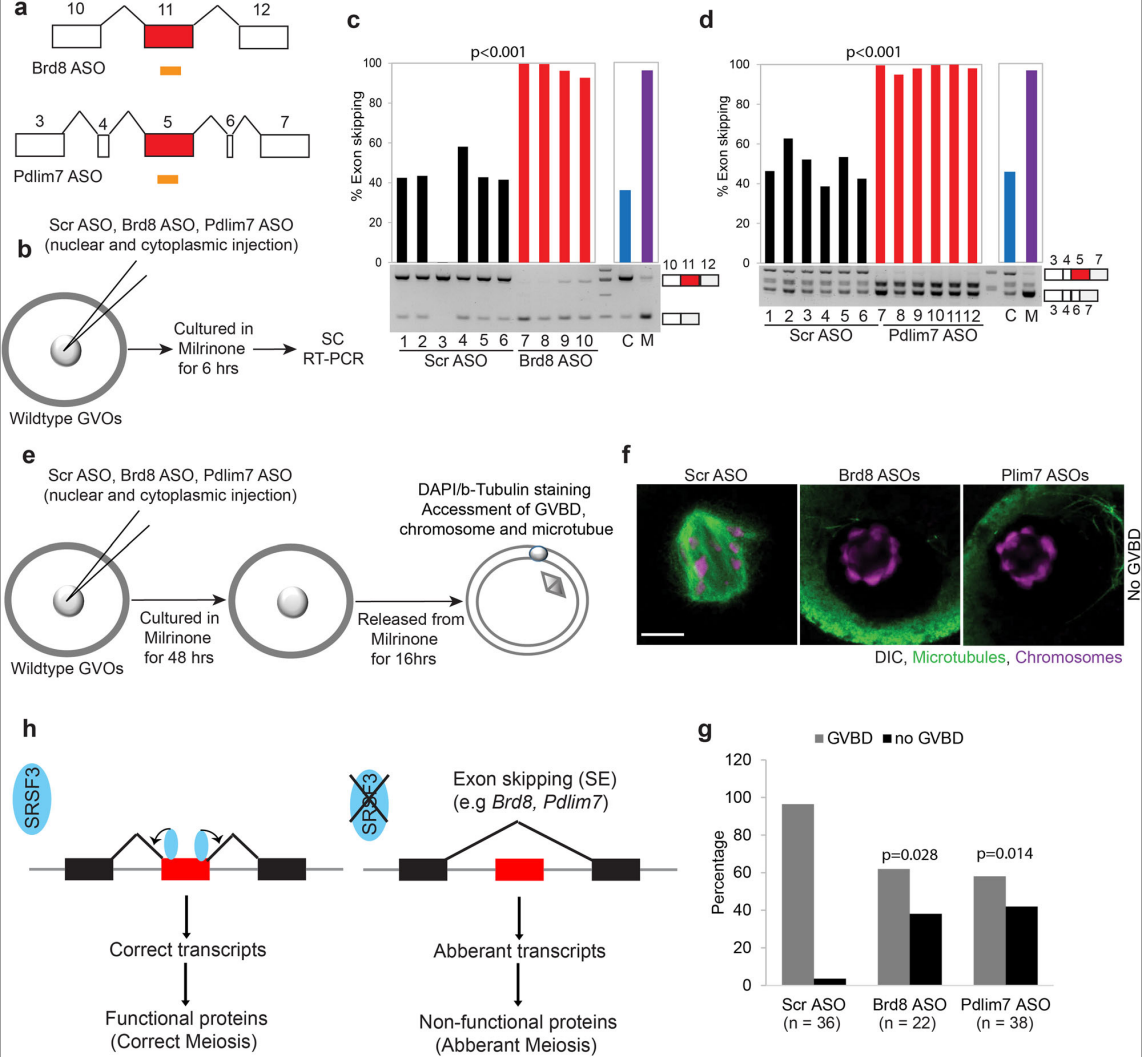
Antisense oligonucleotides generating Brd8- and Pdlim7-alternative splicing recapitulate the GVBD defect in *Srsf3*-mutant oocytes. **a** Schematic representation of ASOs designed to induce exon skipping of *Brd8* and *Pdlim7* in wild-type oocytes. **b** Schematic illustration of an experiment to validate efficacies of ASOs in inducing exon skipping of *Brd8* and *Pdlim7* in wild-type oocytes. **c**, **d** Validation of efficacies of Brd8 (**c**) and Pdlim7 (**d**) ASOs by semiquantitative PCR. Percentage of exon skipping in individual control and mutant oocytes is shown on the top. C: control oocyte, M: mutant oocyte. Mann–Whitney test (Wilcoxon rank sum test) was used to calculate p-value. **e** Schematic illustration of an experiment to validate function of ASOs inducing exon skipping for *Brd8* and *Pdlim7* and GVBD defect in wild-type oocytes. **f** Representative confocal images of wild-type GV oocytes injected with *Brd8* or *Pdlim7* ASOs show no GVBD while oocyte injected with scramble ASO shows normal GVBD. Chromosome in magenta. Microtubule in green. Scale bar, 20 µm. **g** A graph shows percentages of wild-type oocytes undergoing GVBD after injected with Brd8 and Pdlim7 ASOs. *p*-value was calculated by Student's *t*-test. The data were calculated from two independent experiments. **h** Proposed schematic of the role of SRSF3 in regulating alternative splicing in mouse oocytes. Black and red boxes represent exons, red box is alternative spliced exon

Next, we investigated whether artificial induction of AS can recapitulate the GVBD defect in mutant oocytes. We designed steric hindrance antisense oligonucleotides (ASOs)^50^ to target putative SRSF3 binding sites^44^ to induce specific skipping events of *Brd8* exon 11, *Pdlim7* exon 5 and *Npm2* exon 2 and exon 3 (Fig. 4a, Supplementary Figure S4a, Supplementary Table S1). We then microinjected these ASOs and scramble ASOs into cytoplasm and nucleus of mouse wild-type oocytes and performed RT-PCR on single-injected oocytes with primers flanking the skipped exons (Fig. 4b). Our results showed that microinjection of the ASOs into mouse wild-type oocytes switched all three AS events toward the splicing patterns observed in *Srsf3-*mutant oocytes (Fig. 4b–d, Supplementary Figure S4b-c). Next, we allowed ASO-injected oocytes to enter meiosis and examined the effect of ASOs-induced exon skipping on GVBD by immunostaining of tubulin, a microtubule marker and DAPI to visualize chromosome (Fig. 4e, f, Supplementary Figure S4d). We found that ASO-mediated changes in *Brd8* and *Pdlim7* AS caused partial failure in GVBD in these injected wild-type oocytes (Fig. 4g). In contrast, ASO-mediated *Npm2* exon skipping did not affect GVBD (Supplementary Figure S4e). These results indicated that SRSF3-mediated *Brd8* and *Pdlim7* exon inclusion is essential to maintain proper GVBD in mouse oocyte meiosis.

### *Srsf3*-knockout oocytes show derepression of B2 SINE retrotransposon

**Fig. 5:**
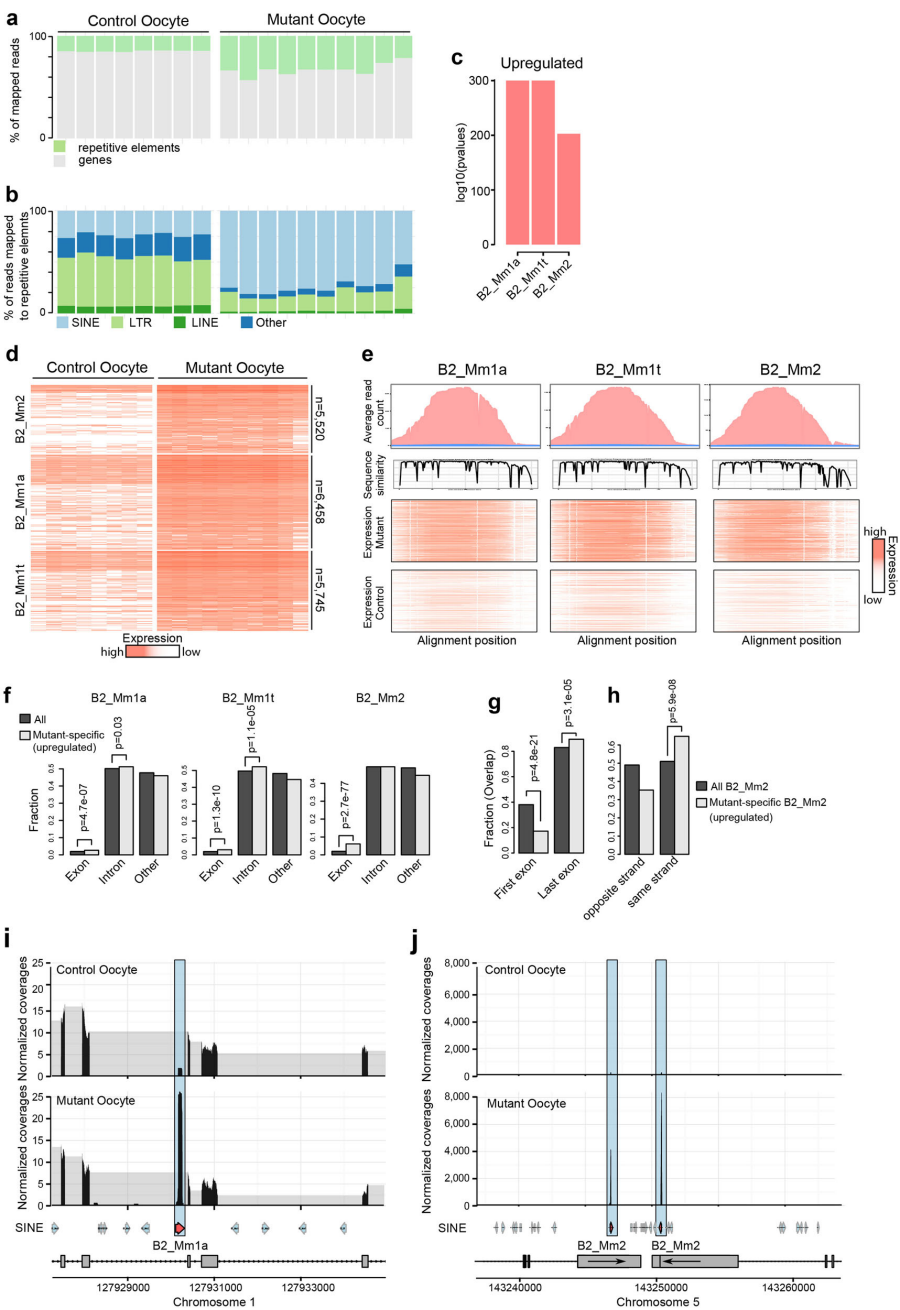
Upregulated expression of B2 SINE classes in *Srsf3* mutant oocytes. **a** Percentage of mapped reads that overlap with repetitive elements for all single control (left) and mutant (right) oocytes. **b** Percentage of reads mapped to different repeat classes (100% = all reads that map to repeats) in control (left) and mutant (right) oocytes. **c** Upregulated retrotransposons in mutant oocytes are significantly enriched in B2_Mm1a, B2_Mm1t, and B2_Mm2 SINE classes. **d** A heatmap shows expression of individual repeat elements from three upregulated B2 SINE classes in control and mutant oocytes. The color indicates the expression level. Numbers of repeat elements are indicated on the right (5520 B2_Mm2 elements, 6458 B2_Mm1a elements, and 5745 B2_Mm1t elements). **e** Expression and sequence similarity of three B2 SINE classes that are upregulated in mutant oocytes. The plot on top panel (plot: average read count) shows the average expression for all elements from these classes. Sequence similarity shows the conservation along the SINE B2 sequences (plot: sequence similarity). The two bottom panels shows the average of mapped reads of all control and mutant samples for each element (plot: expression mutant/expression control, red indicates high number of mapped reads, white indicates low number of mapped reads). **f** Fraction of mutant-specific B2_Mm1a, B2_Mm1t, and B2_Mm2 elements that overlap with exons, introns, or intergenic regions of genes. Significance was estimated using Fisher’s test. **g** Fraction of mutant-specific B2_Mm2 elements that overlap with the first exon or last exon of genes. **h** Fraction of mutant-specific B2_Mm2 elements that overlap with genes on the same strand and opposite strand. **i** Normalized RNA-Seq data for a locus that show increased expression of intronic B2_Mm1a element in mutant oocytes. **J** An example of loci with increased expression of two B2_Mm2 elements that overlap with the last exon of two genes on the same strand

Next we investigated our observation that the number of RNA-Seq reads that could be assigned to genes was smaller in the mutant oocytes compared to the control samples, suggesting that the overall composition of the transcriptome is substantially altered. Surprisingly, this difference could largely be explained by an upregulation of repetitive elements (Fig. 5a). In particular, we found that SINEs, a highly repetitive class of retrotransposons, was systematically upregulated (Fig. 5b). SINE retroelements are ubiquitous, and located throughout their host genome from intergenic regions to being embedded in protein-coding genes^51, 52^, but are usually repressed as in control oocytes to protect against adverse consequences^51^. The prominent upregulation of SINE elements suggests a possible contribution of retrotransposons to the observed GVBD defect in *Srsf3* knockout mice, we therefore further investigated this SINE upregulation.

Among all SINE families, elements from the B2_Mm1a, B2_Mm1t, and B2_Mm2 were significantly over-represented in the set of upregulated elements (*p* < 1e−16, Fig. 5c). Using the uniquely mapped reads, we identified several thousand elements that showed an increase in expression in the mutant oocytes for each of the three SINE families (Supplementary Table S4, Fig. 5d). Genes overlapping such SINE elements showed higher expression in mutant cells, however the differences were much smaller compared to the upregulation of SINE expression (Supplementary Figure S5a-c). To better understand the transcripts that are generated, we first aligned the expressed elements, and then mapped the RNA-Seq reads to the aligned elements (Fig. 5e). Our analysis did not show prominent splicing patterns, suggesting that the generated RNAs largely consist of transcribed SINE elements (Fig. 5e).

To test if the expressed SINE elements have different properties compared to non-expressed elements, we investigated their distribution with respect to annotated genes. We observed an enrichment of mutant-specific elements in introns for the B2_Mm1a and B2_Mm1t families, and an enrichment in exons for elements from all three families (Fig. 5f). This enrichment in exons was particularly strong for the B2_Mm2 family (Fig. 5f). When we investigated exons that overlapped with upregulated SINE elements, we found that they were significantly enriched at the 3′ end (Fig. 5g, j). In addition, expressed elements were more often on the same strand as the overlapping gene (Fig. 5h–j). Using single cell quantitative PCR, we confirmed that B2 SINE elements were upregulated in *Srsf3*-knockout oocytes (Supplementary Figure S5d). B2 SINE elements were also highly expressed in zygotic wild-type16-C embryos and this expression is unaffected in zygotic knockout 16-C embryos (Supplementary Figure S5e). This suggests that the SRSF3 acts to repress SINE element expression specifically in oocytes.

To investigate how SRSF3 regulates B2 SINE expression, we examined the expression of epigenetic modifiers that repress different types of retrotransposon in mouse ES cells and germ cells^53^. These modifiers have important roles in regulating DNA methylation/demethylation, histone methylation, miRNA biogenesis, or piRNA pathway. While there was no significant difference in transcript levels of most retrotransposon repressors, we found a 2-fold downregulation of Piwil1 (also known as Piwi or Miwi) in mutant oocytes compared to controls (Supplementary Figure S5f, S5g), suggesting that aberrant piRNA pathway activity upon SRSF3 depletion might contribute to B2 SINE upregulation. On the other hand, using an online tool (RBPmap) (http://rbpmap.technion.ac.il/)^69^, we found several potential SRSF3 binding sites in expressed B2 SINE elements (Supplementary Figure S5h). In addition, we analyzed published data of CLIP experiments in embryonic carcinoma cells^44^ and found that SRSF3 can indeed bind directly to B2 SINE elements (Supplementary Figure S5i), suggesting that loss of direct binding of SRSF3 to B2 SINE RNAs could contribute to the mutant phenotype as well. Taken together, our analysis identifies a significant change in the transcriptome of oocytes in response to SRSF3 depletion that specifically induces expression of a subset of SINE retrotransposons.

### Upregulation of B2 SINE expression contributes to GVBD defect in *Srsf3*-knockout oocytes

**Fig. 6:**
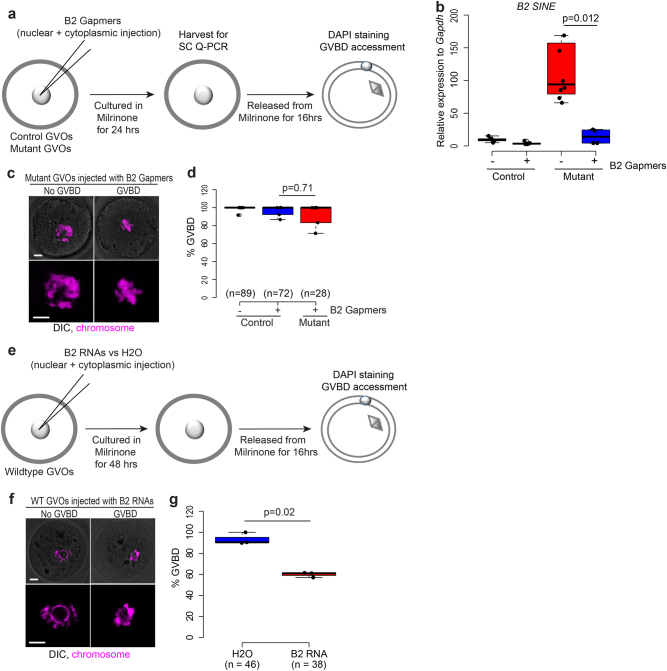
Functional validation of SINE B2 in mouse oocyte meiosis. **a** Schematic illustration. Control and mutant fully grown GV oocytes were injected with a pool of four gapmers targeting the consensus sequence of all B2 SINE elements and cultured in M16 supplemented with Milrinone to prevent GVBD. Oocytes were collected at 24-h postinjection for single cell Q-PCR to measure knockdown efficiency. In different experiments, after 24-h culture in M16 supplemented with Milrinone, injected oocytes were released from Milrinone for 16-h to access GVBD efficiency by DAPI staining. **b** A box plot shows transcript level of B2 SINE in control and mutant oocytes injected with a pooled gapmers targeting B2 SINE sequence. Each black dot represents an individual oocyte. *p*-value is calculated by two-tailed Student’s *t*-test. **c** Representative confocal microscopy of mutant GV oocytes injected with B2 SINE gapmers with or without GVBD. Chromosome in magenta. Scale bar, 20 µm. **d** A box plot shows the percentage of control and mutant oocytes undergoing GVBD after injected with a pooled gapmers targeting B2 SINE sequence. Each black dot represents an individual experiment replicate. *p*-value was calculated by two-tailed Student’s *t*-test. **e** Schematic illustration. Control and mutant oocytes were injected with B2 SINE RNAs. Oocytes were culture in M16 medium supplemented with Milrinone for 48-h and released from Milrinone for 16-h to access GVBD efficiency by DAPI staining. **f** Representative confocal microscopy of wild-type GV oocytes injected with B2 SINE RNAs with or without GVBD. Chromosome in magenta. Scale bar, 20 µm. **g** A box plot shows the percentage of wild-type GV oocytes undergoing GVBD after injected with either H2O or B2 SINE RNAs

To test if the upregulation of B2 SINE elements contributes to the GVBD defect we investigated whether reduction of upregulated B2 SINE expression could rescue the GVBD defect in mutant oocytes. Antisense “gapmer” oligonucleotides that direct RNase-H cleavage of target RNAs have been used to reduce the expression of B2 SINE RNAs^54^. Therefore, we designed four gapmers targeting different parts of the consensus sequence of the upregulated B2 SINE (Supplementary Table S1). To examine the effect of these gapmers on the expression of B2 SINE RNAs, we microinjected the pooled gapmers into control and mutant fully grown GV oocytes and measured the expression of B2 SINE RNAs using single cell quantitative PCR after 24-h culture in vitro (Fig. 6a). Expression of B2 SINE RNAs in mutant oocytes injected with the gapmers was significantly downregulated compared to non-injected oocytes (Fig. 6b). Strikingly, we found that mutant oocytes injected with B2 SINE gapmers underwent GVBD similar to control oocytes, and control oocytes injected with B2 SINE gapmers (Fig. 6c, d). These results suggest that reduction of upregulated B2 SINE RNAs can indeed rescue the GVBD defect in mutant oocytes.

Upregulation of retrotransposons is associated with increased nuclear double-strand breaks (DSBs) and GVBD defect in mouse *Marf1-*depleted oocytes^1^. However, we found no significant difference in the numbers of DSBs between control and mutant oocytes (Supplementary Figure S6a-b), suggesting that upregulated B2 SINE may not cause GVBD defect by increasing DSBs. To examine whether overexpression of B2 SINE RNAs directly cause GVBD defect in mouse wild-type oocytes, we synthesized RNAs from consensus sequences of B2 SINE elements using in vitro transcription. We then microinjected B2 RNAs into wild type fully grown GV oocytes and measured the percentage of injected oocytes which had undergone GVBD (Fig. 6e). We found that oocytes injected with B2 SINE RNAs showed a significant reduction in the percentage of GVBD (40% reduction) as compared to those injected with water (Fig. 6f, g). These results indicate that upregulation of B2 SINE RNAs contributes to the GVBD defect in mutant oocytes. Together our study highlights how precise control of the maternal transcriptome integrity is essential to facilitate the completion of oocyte meiosis and early embryo development.

## Discussion

The maternally inherited factors in oocytes are essential for the development of fertilization-competent oocytes and embryogenesis. However, it has remained unclear how the establishment of the maternal transcriptome is controlled and what the critical regulators are. A number of lines of evidence have suggested that alternative pre-mRNA splicing and processing make an important contribution to shaping the maternal transcriptome^10, 12^ Here we report that the RNA-binding protein SRSF3 is a key factor that contributes to the precise establishment and regulation of the maternal transcriptome. The most prominently observable phenotype of SRSF3 depletion is a defect in GVBD that causes sterility in *Srsf3* maternal knockout mice (Fig. 1). Single cell RNA-Seq analysis of control and *Srsf3* knockout oocytes (Fig. 2), revealed that not only does SRSF3 shape the oocyte maternal transcriptome via its expected role in the regulation of AS (Fig. 3, Supplementary Figure S6c), but it has an unanticipated role in suppressing transposable element expression in mouse oocytes (Fig. 5, Supplementary Figure S6c).

A prominent consequence of SRSF3 depletion on AS was increased skipping of both annotated cassette exons (e.g., in *Brd8* and *Pdlim7*), as well as exons that have not previously been observed to be skipped (e.g., in *Npm2*) (Fig. 3). Moreover, integration of our RNA-Seq data with published SRSF3 iCLIP data^44^ showed a peak of SRSF3 binding within these exons (Supplementary Figure S3e), consistent with conventional action of SRSF3 as a splicing activator that acts by binding to exon splicing enhancers. Recently SRSF3 was shown to co-regulate some alternatively spliced exons in conjunction with the nuclear m6A “reader” protein YTHDC1^55^. Whether SRSF3 is involved in a similar functional cross-talk between the epitranscriptome and AS in mouse oocytes remains an interesting possibility.

The SRSF3-dependent AS events included exons within genes likely to be associated with the GVBD phenotype. Strikingly, although numerous AS events were affected by SRSF3 depletion, we were able to partially reproduce the GVBD defect using exon-targeting ASOs to induce alterations in individual splicing events in the *Pdlim7* or *Brd8* genes (Fig. 4). These AS events were selected due to the functional association of the genes with GVBD. Nevertheless, the precise effects of the AS events on protein isoform function is not clear. Skipping of *Brd8* exon 11 maintains reading frame and is remote from the Bromodomain encoded by mRNA sequence from exon 17 to exon 19. Switching between selection of *Pdlim7* exons 5 and 6 also maintains reading frame, affecting the encoded protein immediately on the C-terminal side of the PDZ domain, and upstream of the C-terminal LIM domain. Although, the exon 5 encoded insert is substantially longer (40 vs. 6 amino acids), the exon 6 isoform, which is induced upon *Srsf3* knockout, encodes a PFAM domain of unknown function (DUF4749)^56^, which might modulate the actin binding function of the PDZ domain. In contrast, skipping of *Npm2* exon 2 and exon 3 is clearly predicted to lead to loss of function by frameshifting leading to NMD, but manipulation of this event had no effect upon GVBD (Supplementary Figure S4).

Surprisingly, loss of SRSF3 in oocytes also dramatically changed the composition of the transcriptome with a prominent and consistent surge in B2 SINE retrotransposon expression (Fig. 5). Upregulation of expressed Alu sequences was observed in human cells upon knockdown of hnRNPC as a result of “exonization” of intronic Alu elements^57^. The Alu elements contain sequences resembling bona fide splice sites, but these are usually blocked by hnRNPC. In contrast the upregulated B2 SINES in *Srsf3* knockout oocytes are autonomously transcribed (Fig. 5). Strikingly, experimental overexpression of B2 SINEs in wild-type oocytes was sufficient to induce a defect in GVBD (Fig. 6e, f), indicating that multiple changes in the transcriptome contribute to the observed phenotype of *Srsf3* knockout oocytes. In contrast to splicing, where a direct effect of SRSF3 is highly likely (Fig. 4h), the mechanism that leads to induction of B2 SINEs and subsequent defects in GVBD is not evident, but could plausibly arise from indirect or direct actions of SRSF3. Upregulation could be an indirect effect caused by downregulation of components of the PIWI-interacting RNAs (piRNA) pathway, such as Piwil1 (Supplementary Figure S5e, f), leading to loss of silencing or degradation of B2 SINE retrotransposons. We did not detect altered splicing of Piwil1, which might account for its downregulation via a direct splicing effect of SRSF3. However, this could be related to lack of sequence depth in our single cell RNA-Seq data, especially if a novel exon skipping event resulted in NMD. In this scenario, B2 SINE upregulation would be a downstream consequence of disrupting the conventional function of SRSF3 as a splicing factor.

On the other hand, direct effects might be possible as well, as the B2 SINE consensus sequence contains predicted SRSF3 binding sites and bioinformatics analysis of published SRSF3 CLIP data demonstrated that SRSF3 binds directly to B2 SINE (Supplementary Figure S5G–I). Such direct action would imply that binding of SRSF3 leads to degradation of B2 SINE RNAs, which would represent a novel function for SRSF3. Binding of SRSF3 by B2 SINE might also explain the ability of injected B2 SINE RNA to induce a GVBD phenotype. Sequestration of SRSF3 by the injected RNA could lead to misregulation of target AS events due to lower available levels of SRSF3, in a process similar to the misregulated splicing caused by sequestration of muscle blind-like RNA binding proteins by CUG expansion RNA in myotonic dystrophy^58^.

In addition to its well-known role as a splicing regulator, SRSF3 has also been documented to play transcript-specific roles in RNA polyadenylation^21^, RNA export^22^, pri-miRNA processing^23^, and translation^24^. It remains possible that misregulation of some of these processes might also contribute to the GVBD phenotype. Nevertheless, the ability to partially recreate the GVBD phenotype by manipulation of individual SRSF3-regulated AS events is consistent with this being the major SRSF3 function that is disrupted.

Computational and experimental limitations pose challenges to identifying a molecular mechanism in oocytes, as the possible experimental assays are limited to those that were developed for single cells. Computationally, one of the major challenges in studying B2 elements is their short and highly repetitive DNA sequence. Even though a large number of active loci can be identified, they cannot always be distinguished, and therefore the expression estimates for individual elements are more uncertain compared to estimates for gene expression. Our analysis indicates that the expressed B2 SINE elements are distinct from the non-expressed ones with respect to existing genes (Fig. 5), however, a high level of uncertainty makes it challenging to generate accurate bioinformatics predictions about any association with expression of the surrounding genes. While research on repression of retrotransposons has provided some insights into the role of DNA methylation, histone modifications, and sequence-specific RNA degradation through endogenous small interfering RNAs or piRNAs^52^, research examples about such a strong induction of B2 SINE expression in oocytes are not known. Even though the mechanism remains unclear, our findings open exciting new possibilities of regulation of retrotransposon through context-specific RNA binding proteins.

In summary, we report a highly reproducible, pervasive change in transcriptome composition, together with the dysregulation of many hundreds splice isoforms after knockout of *Srsf3* in mice oocytes. Our study highlights the relevance of AS and retrotransposon expression in maintaining cellular function, and suggest a prominent role of SRSF3 in the establishment and control of transcriptional integrity in mouse oocytes.

## Material and methods

### Mouse strain and genotyping

*Srsf3*^tm1Pjln^ mice (*Srsf3*^flox/flox^) are kindly provided by Nielsen and colleagues^25^. *Srsf3*^flox/flox^ mice were crossed with *Zp3-Cre* transgenic mice carrying cre-recombinase under the control of the oocyte-specific *Zp3* promoter^26^ to generate *Zp3-Cre*; *Srsf3*^flox/+^ male mice. These mice were then backcrossed to *Srsf3*^flox/flox^ female mice, and *Zp3-Cre; Srsf3*^flox/flox^ female were selected (Supplementary Figure S1c). Oocytes from these females are SRSF3-deleted (Supplementary Figure S1d). Genotyping was done by PCR using DNA extracted from tails tips of 14-day-old mice. The primer pairs used to detect the presence of the *Zp3-Cre* transgene and the *Srsf3*-floxed alleles were as described^25, 26^.

### Collecting oocytes and preimplantation embryos

C57BL/6 or B6CBAF1/J (F1) female mice were superovulated by injecting pregnant mare’s serum gonadotropin (PMSG), followed by human chorionic gonadotropin (hCG) after 48 h and then mated with male mice. MII oocytes and zygotes were collected from oviducts 17–22 h after hCG injection. Cumulus cells were removed by incubation with 0.3 mg/ml hyaluronidase (Sigma, H4272) in M2 medium. The embryos were recovered in M2 medium and cultured in M16 medium in BD Falcon Organ Culture Dish in 5% CO_2_ at 37 ° C. The embryos were collected at different stages for immunostaining. Alternatively, embryos from the 2-cell to 8-cell stage were flushed from oviducts at 1.5 and 2.5 dpc and embryos from blastocyst stage were flushed from the uterus at 3.5 dpc.

GV oocytes were obtained from ovaries of female mice. Ovaries were placed in a Petri dish with prewarmed (37 °C) M2 medium (Sigma) supplemented with Milrinone (Sigma) to prevent oocytes undergoing GVBD. GV oocytes were released by puncturing antral follicles with a 30G needle. Cumulus cells were removed by passing follicles through a glass mouth pipette with a small open-end. For in vitro maturation, oocytes were washed and cultured in Milrinone-free M16 medium (Millipore) for various period of times in 5% CO_2_ at 37 °C.

### Expression constructs, RNA synthesis, and oligonucleotide design and synthesis

The coding sequences of human H2B-RFP, EB3-mEGFP^59^, and the FRET biosensor specific for CyclinB1-CDK1^28^ were cloned into pBluescript RN3P^60^ and verified by DNA sequencing. mRNAs were transcribed in vitro using the mMessage mMachine T3 kit (Ambion). RNA quality and concentration were quantified by Tapstation and Nanodrop.

Novel ASOs were applied to bind to nascent transcripts of a target gene via Watson–Crick bonding, to exert steric hindrance effects against splicing factors to modulate splicing. Novel gapmers were applied to bind B2 SINE RNAs to elicit RNase-H mediated degradation. All the ASOs are synthesized as single-stranded 2-*O*-methyl modified RNA bases linked by a phosphorothioate backbone (IDT, UK). The sequences of ASOs are 5′-CGGUGUGUGUAUCAUUCUCUAGUGU-3′ for scramble ASO; 5′-GUGAAGGAAGGAAGAGGA GUGGUGAACUGUGUG-3′ for *Brd8*_exon 11 ASO; 5′-CUUGGUGCAAAAGUGUACCUCGGG GG-3′ for *Pdlim7*_exon 5 ASO; 5′-UGUUGUGGGGAAAGAUUAUGUCUGUGGUG-3′ for *Npm2*_exon 2 ASO and 5′-AAAGGUGCAAGUCUGCUUUUCCUGAUUGAGUU-3′ for *Npm2*_exon3 ASO. Gapmers are synthesized as single-stranded DNA bases each flanked with three 2 -O- methyl modified RNA bases linked by a phosphorothioate backbone (IDT, UK). The sequences of B2 gapmers are 5′-UUC(dA)(dA)(dA)(dT)(dC)(dC)(dC)(dA)(dG)(dC)(dA)(dA) (dC)(dC)(dA)(dC)(dA)(dT)(dG)(dG)(dT)(dG)(dG)(dC)(dT)(dC)(dA)(dC)(dA)ACC-3′; 5′-AGU (dT)(dC)(dA)(dA)(dA)(dT)(dC)(dC)(dC)(dA)(dG)(dC)(dA)(dA)(dC)(dC)(dA)(dC)(dA)(dT)(dG)(dG)(dT)(dG)GCU-3′; 5′-GAG(dT)(dT)(dC)(dA)(dA)(dA)(dT)(dC)(dC)(dC)(dA)(dG)(dC)(dA)(dA) (dC)(dC)(dA)(dC)AUG-3; 5′-AGC(dA)(dA)(dC)(dC)(dA)(dC)(dA)(dT)(dG)(dG)(dT)(dG) (dG)(dC)(dT)(dC)(dA)(dC)(dA)ACC-3′. The ASOs and gapmers (Supplementary Table S1) were rationally designed for optimal efficiency, as previously described^50^. Briefly, target sites were selected by a computational algorithm that accounted for co-transcriptional binding accessibilities, binding thermodynamics, and presence of regulatory motifs.

### Oocyte microinjection

GV oocytes were maintained in M2 medium supplemented with Milrinone during the course of microinjection. We used Femtojet 4i system (Eppendorf) and injecting setting is Pi = 110 hpA, Pc = 15 hpA, and ti = 0.5 s to deliver volumes ranged from 10–15 pl into GV and/or cytoplasm of oocytes. For live imaging, we injected EB3-mEGFP RNA at concentration of 300 ng/µl and H2B-RFP RNA at concentration of 10 ng/µl into oocyte cytoplasm. For FRET experiment, we injected FRET sensor at concentration 300 ng/µl and H2B-RFP at concentration 10 ng/µl. For functional analysis of B2 RNAs, we injected gapmers targeting B2 RNAs at concentration of 100 µM into oocyte GV and cytoplasm. For functional analyses of AS, we injected 100 µM of ASOs into oocyte GV and cytoplasm. Sequence of ASOs and gapmers are provided in Supplementary Table S1.

### Live imaging, FRET experiment, and image analysis

To facilitate oocyte handling and high-resolution imaging we have developed a multi-well glass chip that contains an array of 252 well-chambers open to the medium. This device was developed in collaboration with Dolomite (The Dolomite Centre Ltd), a microfluidics device manufacturer (http://www.dolomite-microfluidics.com/webshop/microfluidic-chips-wellplate-chips-c-5_159/embryo-immobilization-chip-p-908) (Dolomite part number: 3200208). The chip is bonded to a glass reservoir to hold medium. To mount the device onto the microscope it was inserted into a metal interface (Dolomite part number 3200209) compatible with standard 35 mm petri-dish microscope stage inserts.

Time-lapse imaging was carried out on a confocal spinning-disk microscope system (Intelligent Imaging Innovations, Inc. 3i), comprising an Observer Z1 inverted microscope (Zeiss), a CSU X1 spinning-disk head (Yokogawa) and a QuantEM 512SC camera (Photometrics). All data shown were collected using a 63 × NA 1.2 w.corr. objective (Zeiss). Image acquisition and processing was carried out in Slidebook 5 software; Image J was used for additional image processing.

FRET data was analyzed by loading the transmission and donor channel data (*T*(*t*) and *D*(*t*) respectively) and their corresponding background levels (*δT*(*t*) and *δD*(*t*)) into commercially available data analysis software ORIGIN 9.1 (OriginLab). The background levels were subtracted from each channel and the resulting signal normalized to have a maximum and minimum corresponding to 1 and 0 respectively. The background subtracted transmission signal was divided by the background subtracted donor signal to give the FRET signal *F*(*t*),

$$F(t) = \frac{{\left\langle {T(t) - \delta T(t)} \right\rangle _0^1}}{{\left\langle {D(t) - \delta D(t)} \right\rangle _0^1}},$$

where $\left\langle {...} \right\rangle _0^1$ is the contents normalized with a maximum value of 1 and a minimum of 0.

### Immunostaining of oocytes and preimplantation embryos

GV oocytes, MII oocytes, and preimplantation embryos were fixed in 4% paraformaldehyde in PBS for 15 min at room temperature. They were washed in PBS with 0.1% Triton three times and permeabilized with 0.25% Triton in PBS for 1 h at room temperature. The embryos were incubated with primary antibody overnight at 4 °C, after blocking in PBS with 0.1% Triton, 10% FBS and 5% BSA for 1 h at room temperature. Primary antibodies used in this study was α-SRSF3 (a kind gift from Dr Nielsen, 1:100), α-H3S10P (Abcam, 1:100), α-gamma-H2AX (Millipore, 1:100), α-H3K9me3 (Abcam, 1:100), α-H3K9me2 (Abcam, 1:100) and α-H3K9ac (Abcam, 1:100). The oocytes and embryos were then stained with secondary antibodies conjugated with Alexa Fluor 488 or 596 for 1 h at room temperature. DAPI was used for nuclear staining. The embryos were analyzed by Leica Sp5 confocal microscopy using a ×40 oil immersion lens. Images were taken every 2 µm through the embryo.

### Quantitative real-time PCR or semiquantitative PCR for single oocytes or embryos

cDNA from single oocyte or single 16-C embryo was prepared according to previous published protocol^70^. Briefly, a single oocyte or single 16-C embryo was picked and lysed in the buffer comprising 0.9X PCR buffer II (without MgCl2), 1.35 mM MgCl_2_, 0.45% NP40, 4.5 mM DTT, 0.18 U/μl SUPERase-In, 0.36 U/μl RNase inhibitor, 12.5 nM UP1 primer, 0.045 mM dNTP mix. The mRNAs in the lysate were then reverse-transcribed into cDNAs by poly(T) primer with anchor sequence (UP1) by incubating at 50 °C for 30 min, and reverse transcriptase was inactivated by incubation at 70 °C for 15 min. After this, the nonreactive primers were digested by exonuclease I. A poly(A) tail was then added to the first-strand cDNAs at the 3′ end by terminal deoxynucleotidyl transferase. Next, the second-strand cDNAs were synthesized by poly(T) primer with another anchor sequence (UP2). Then these cDNAs were evenly amplified by PCR of 20 cycles of 95 °C for 30 s, 67 °C for 1 min, 72 °C for 6 min (plus 6 s more after each cycle). cDNA was diluted ten time and used for quantitative real-time PCR and semiquantitative PCR. Quantitative real-time PCR was performed using SyberGreen master mix (Sigma) and QuantStudio™ 6 Flex Real-Time PCR System (Life Technology). Semiquantitative PCR was performing using HotStarTaq Master Mix Kit (Qiagen).

### Single cell RNA sequencing

GV oocytes were obtained from ovaries of 3-week-old control and mutant female mice. The zona pellucida was removed using Tyrode’s solution (Sigma). Zona-free oocytes were washed through sterile M2 and 1X PBS-BSA and then placed into individual tubes containing 2 μl of 0.2% Triton X-100 (Sigma) supplemented with 1 U/μl RNAsIN (Ambion). mRNA from the single cells was amplified using the SMARTSeq2 protocol^71^, with the additional inclusion of ERCC spike-in control (1:1,000,000 dilution). Multiplex sequencing libraries were generated from amplified cDNA using Nextera XT (Illumina) and sequenced on a HiSeq 1500 at 100 bp paired end.

### Bioinformatics analysis

RNA-Seq data were mapped against the mouse genome version mm10 with TopHat2-2.0.12^61^ using the annotations of Ensembl version 81^62^. SAMtools^63^ was used to extract uniquely mapped reads from bam files (fgrep –w NH:i:1). R 3.1.2 and Bioconductor 3.0 were used for the RNA-Seq analysis. Repeat was extracted using from the UCSC Genome Browser. Reads were counted using the R package GenomicAlignments^64^ (mode = “Union,” inter.feature = FALSE), only primary read alignments were retained. Reads were only counted in regions not overlapping with repetitive elements. Differentially expressed repeats were defined by using two criteria. Log2FoldChange of these elements should be higher than 1 and the adjusted *p*-values should be lower than 0.05. *P*-values were calculated using the Wilcoxon test and they were adjusted for multiple testing according to Benjamini & Hochberg. Significance for enrichment of families was estimated using Fisher’s test. Repeat alignments were generated using MAFFT v7.154b^65^.

For the junction read count analysis, we first counted all split reads across all datasets. We then annotated these reads according to overlapping known exons. Junction read count ratios were calculated using DESeq2 per gene^66^.

For RNA splicing map, SRSF3 iClip data were collected from Änkö et al.^44^ following procedures described elsewhere^67^. Genome mapping was performed against mouse genome version NCBI37/mm9 using Bowtie (0.12.7). We used the random barcode to discriminate and eliminate PCR duplicates that derived from the original cDNA. Xlinks, peak calling analysis and groups were done at iCount iCLIP analysis web site (http://icount.biolab.si/). Xlinks sites were assessed on differentially spliced exon–intron boundaries. RNA maps were computed as the sum of cDNAs tags that match positions and averaged by the number of splice events on 50 nt bins^68^. Density of xlinks was plotted using geom area from ggplot2 package and smoother with gaussian method. Data analysis was performed using R-3.3.1 (R Core Team). The R packages ggplot2 (2.1.0), latticeextra (0.6.28), reshape2 (1.2.2), and the package GenomicRanges (1.22.4) were used throughout the analysis.

### Statistical analysis

Two-tailed unpaired Student’s *t*-tests and Mann–Whitney test (Wilcoxon rank sum test) were performed on R 3.4.1 program. *P*-value <0.05 were considered as statistically significant. Statistical methods for the RNA-Seq anlaysis are described in the respective section. Briefly, *P*-values were calculated using the Wilcoxon test and they were adjusted for multiple testing according to Benjamini & Hochberg. Significance for enrichment of families was estimated using Fisher’s test.

## Electronic supplementary material

Supplementary Information

Supplementary Table S1

Supplementary Table S2

Supplementary Table S3

Supplementary Table S4

Movie S1_A representative movie showing normal meiosis in control oocyte

Movie S2_A representative movie showing no GVBD in mutant oocyte_1

Movie S3_A representative movie showing delayed GVBD in mutant oocyte_2
