# Supplementary material for: SRSF3 maintains transcriptome integrity in oocytes by regulation of alternative splicing and transposable elements
Source: Cell Discov. 2018 Jun 19;4:33. doi: 10.1038/s41421-018-0032-3 (PMC6006335; doi:10.1038/s41421-018-0032-3)
Supplement: Supplementary file 1 — Supplementary Information [file 41421_2018_32_MOESM1_ESM.pdf]

Supplementary information

1. Supplementary Figure S1 (related to Figure 1)
2. Supplementary Figure S2 (related to Figure 3)
3. Supplementary Figure S3 (related to Figure 3)
4. Supplementary Figure S4 (related to Figure 4)
5. Supplementary Figure S5 (related to Figure 5)
6. Supplementary Figure S6 (related to Figure 6)
7. Supplementary Movie S1 (related to Figure 1)
8. Supplementary Movie S2 (related to Figure 1)
9. Supplementary Movie S3 (related to Figure 1)
10. Supplementary Table S1
11. Supplementary Table S2 (related to Figure 2)
12. Supplementary Table S3 (related to Figure 3)
13. Supplementary Table S4 (related to Figure 5)

Supplementary Figure S1

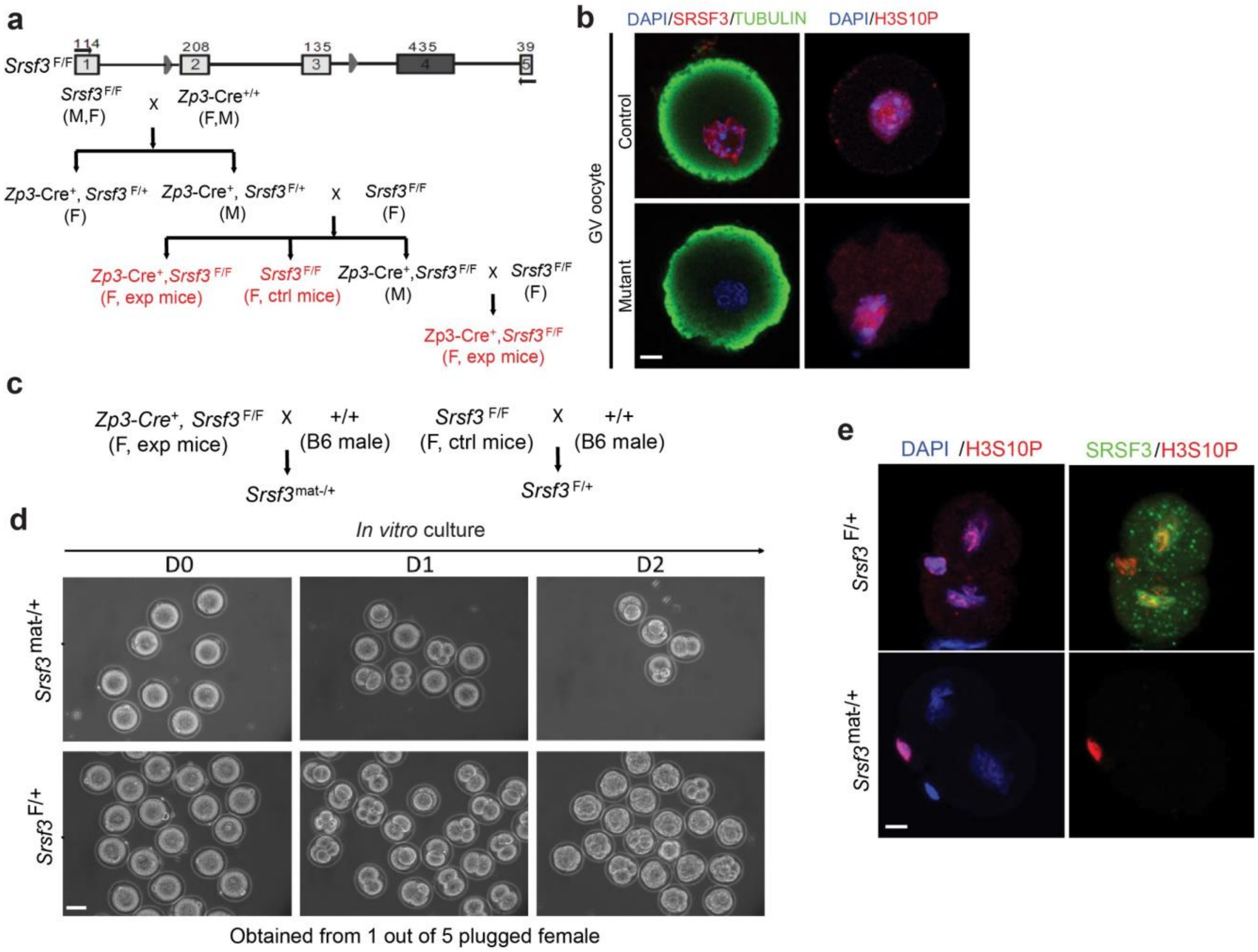

Supplementary Figure S1: Embryos lacking maternal SRSF3 fail to develop beyond 2-cell stage

a. Scheme shows the generation of maternal *Srsf3*-knockout mice (*Zp3-Cre*$^{+}$, *Srsf3*$^{F/F}$)
b. Immunostaining shows loss of SRSF3 protein in maternal *Srsf3* knockout oocytes. Scale bar: 100 µm
c. Scheme shows the generation of preimplantation embryos depleted with maternal SRSF3 protein
d. Maternal *Srsf3*-knockout embryos are arrested at zygote or 2-cell stage. Scale bar: 10 mm
e. Immunostaining shows loss of H3S10P protein in the nucleus of maternal *Srsf3* knockout 2-cell embryos. Scale bar: 100 µm

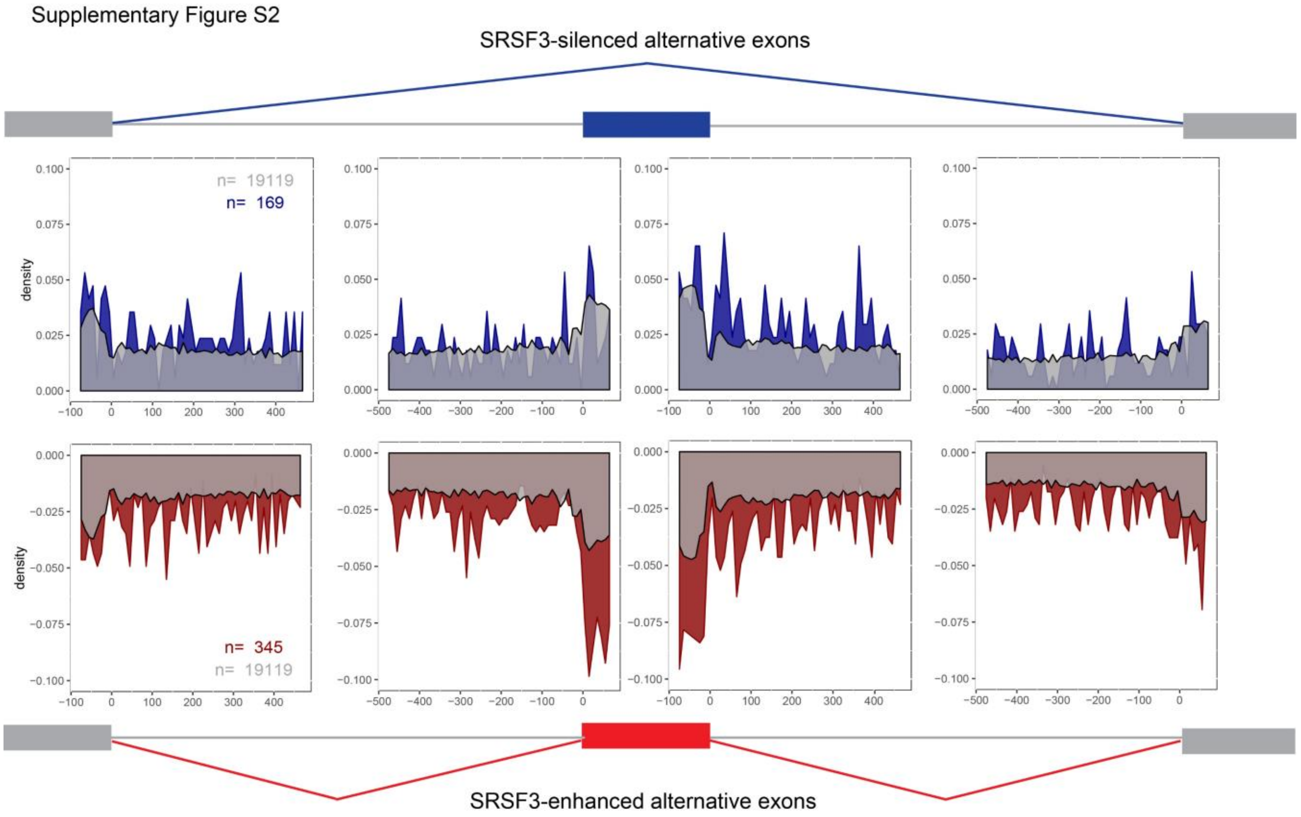

Supplementary Figure S2: a RNA-binding map of SRSF3

SRSF3 crosslinking in SRSF3-regulated pre-mRNAs where position of crosslinked nucleotides was mapped onto the regulated exon and the 500 nucleotides upstream and downstream of its 3'ss and 5'ss, respectively, the upstream flanking exon and 500 nucleotides downstream of its 5'ss and the downstream flanking exon with 500 nucleotides upstream of its 3'ss. The iCLIP tags were mapped onto SRSF3-silenced ASE (blue, n=169), enhanced ASE (red, n=345) and control ASE (grey, n=19119)

Supplementary Figure S3

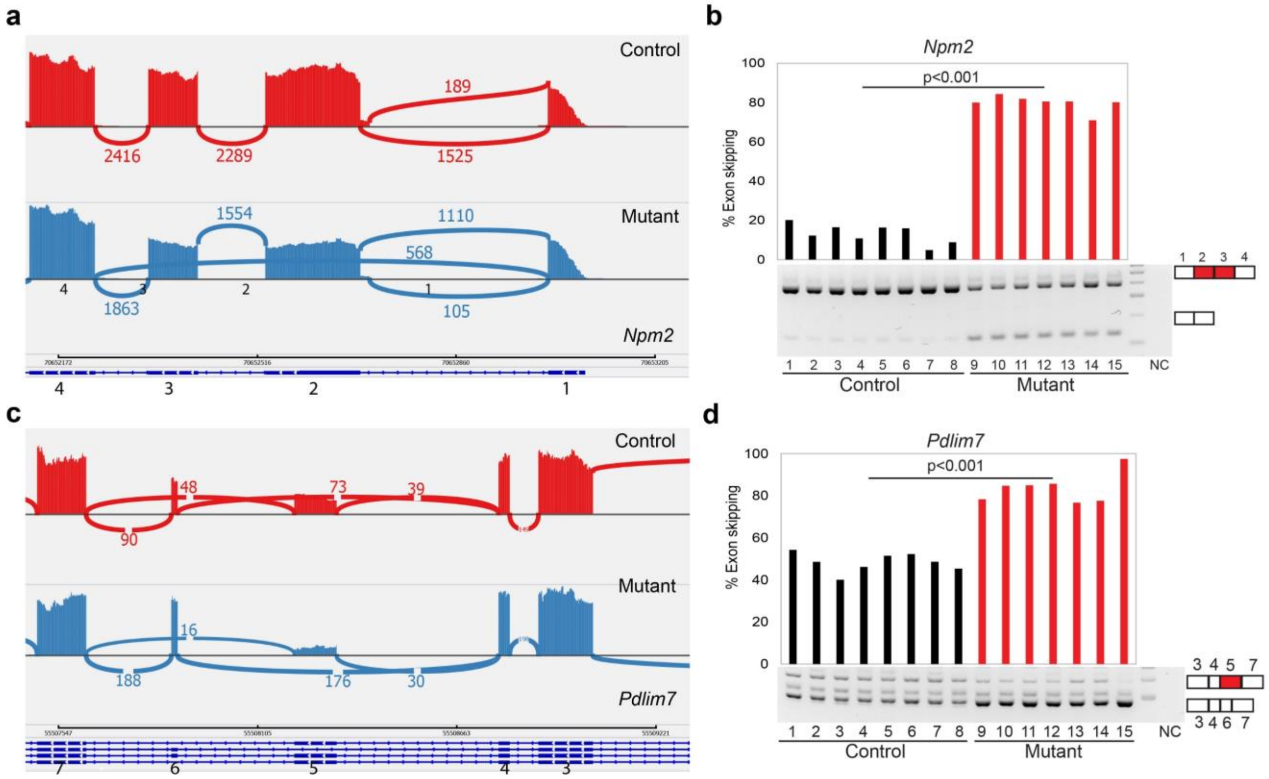

Supplementary Figure S3: Validation of exon skipping events in mutant oocytes
(a,c) Sashimi plots show exon skipping events in *Npm2* and *Pdlim7* transcripts in control and mutant oocytes
(b,d) Validation of the exon skipping events on *Npm2* and *Pdlim7* transcripts in control and mutant oocytes by semi-quantitative PCR. Percentage of exon skipping calculated for control and mutant oocytes is shown on the top. Mann Whitney test (Wilcoxon rank sum test) was used to calculate p-value

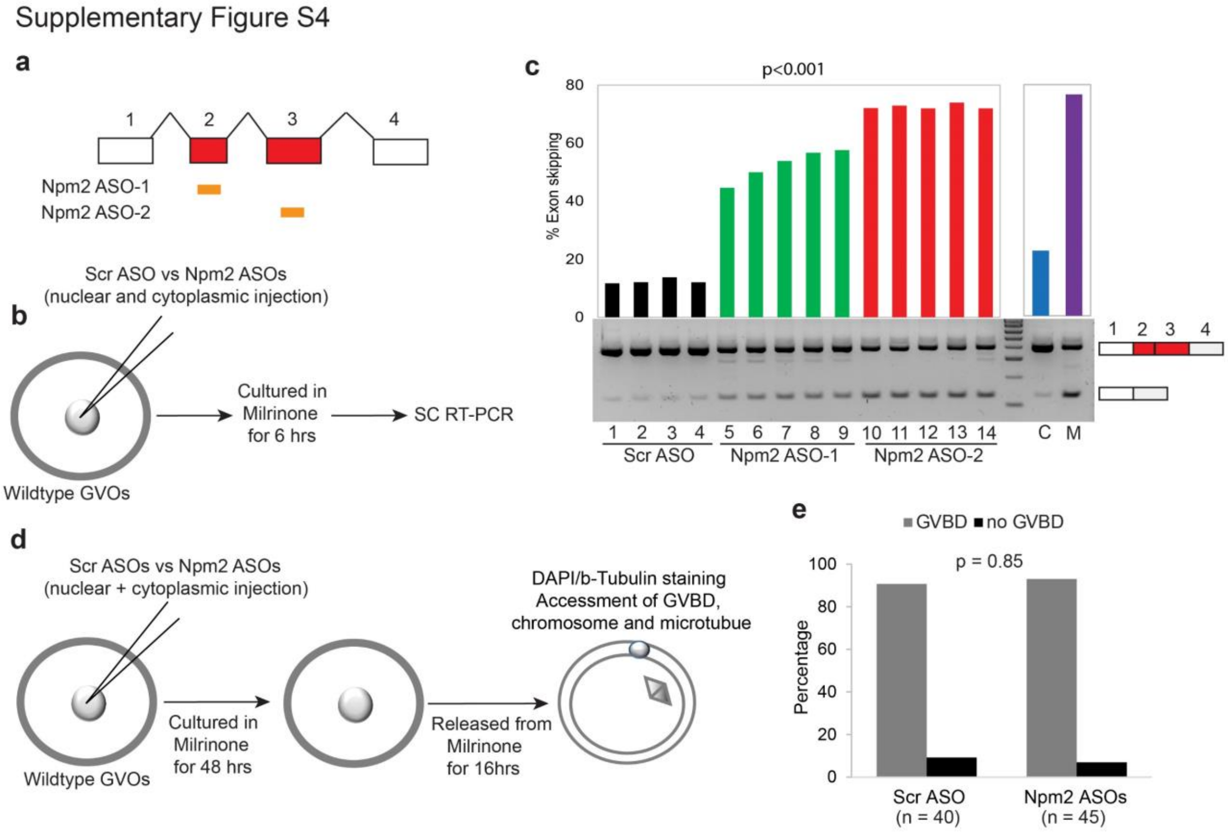

Supplementary Figure S4: Antisense oligonucleotides targeting *Npm2*-alternative splicing do not cause GVBD defect in wildtype oocytes.

a. Schematic representation of ASOs designed to induce exon skipping of *Npm2* in wildtype oocytes
b. Schematic illustration of an experiment to validate efficacies of *Npm2* ASOs in wildtype oocytes
c. Validation of efficacies of *Npm2* ASOs by semi-quantitative PCR. Percentage of exon skipping of *Npm2* in individual control and mutant oocytes is shown on the top. C: control oocyte, M: mutant oocyte. Mann Whitney test (Wilcoxon rank sum test) was used to calculate p-value
d. Schematic illustration of an experiment to validate function of *Npm2* ASOs and GVBD defect in wildtype oocytes
e. A graph shows percentage of wildtype oocytes undergoing normal GVBD after injected with *Npm2* ASOs. p-value is calculated by Student's t-test. The data is calculated from two independent experiments

Supplementary Figure S5

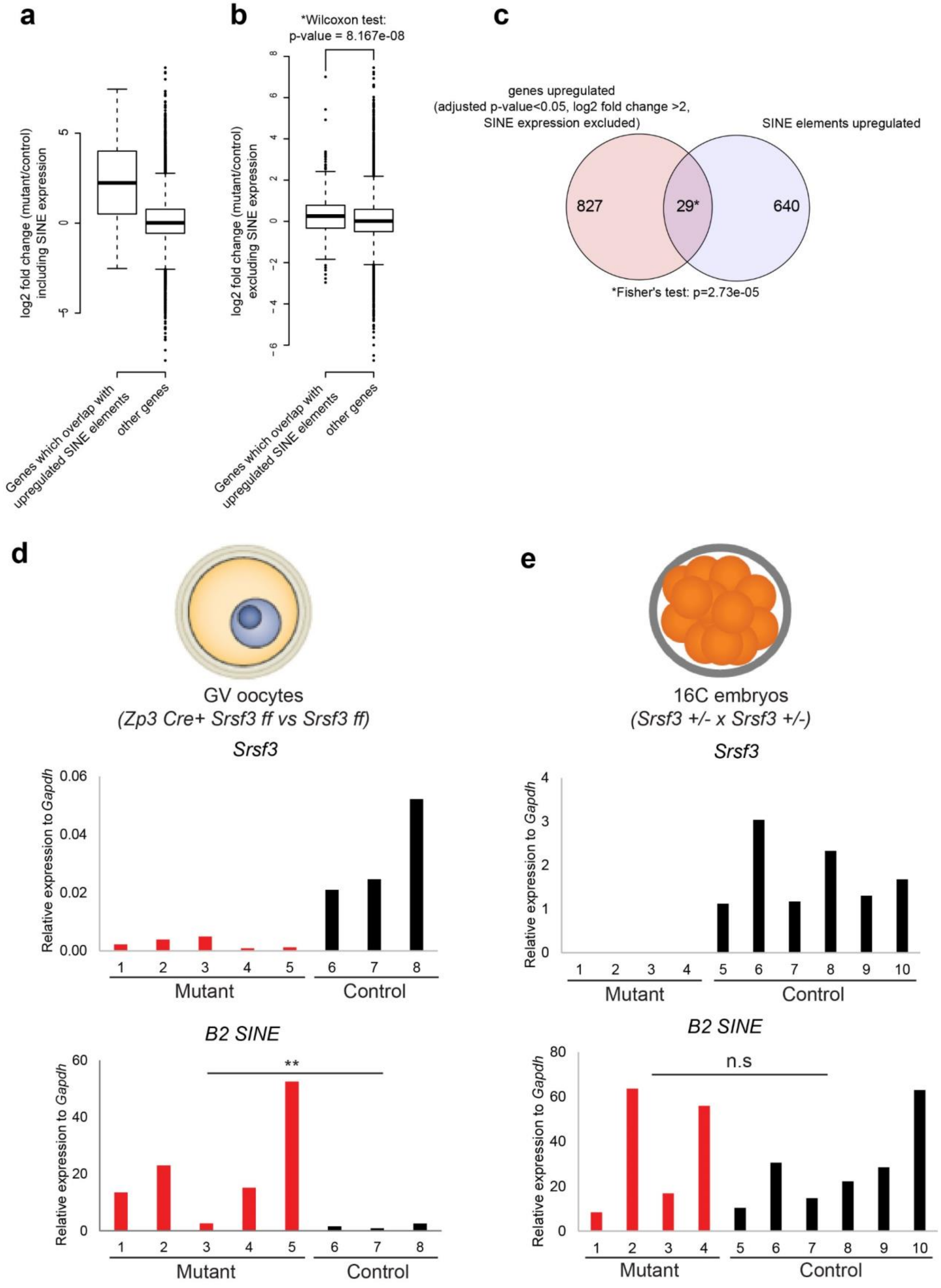

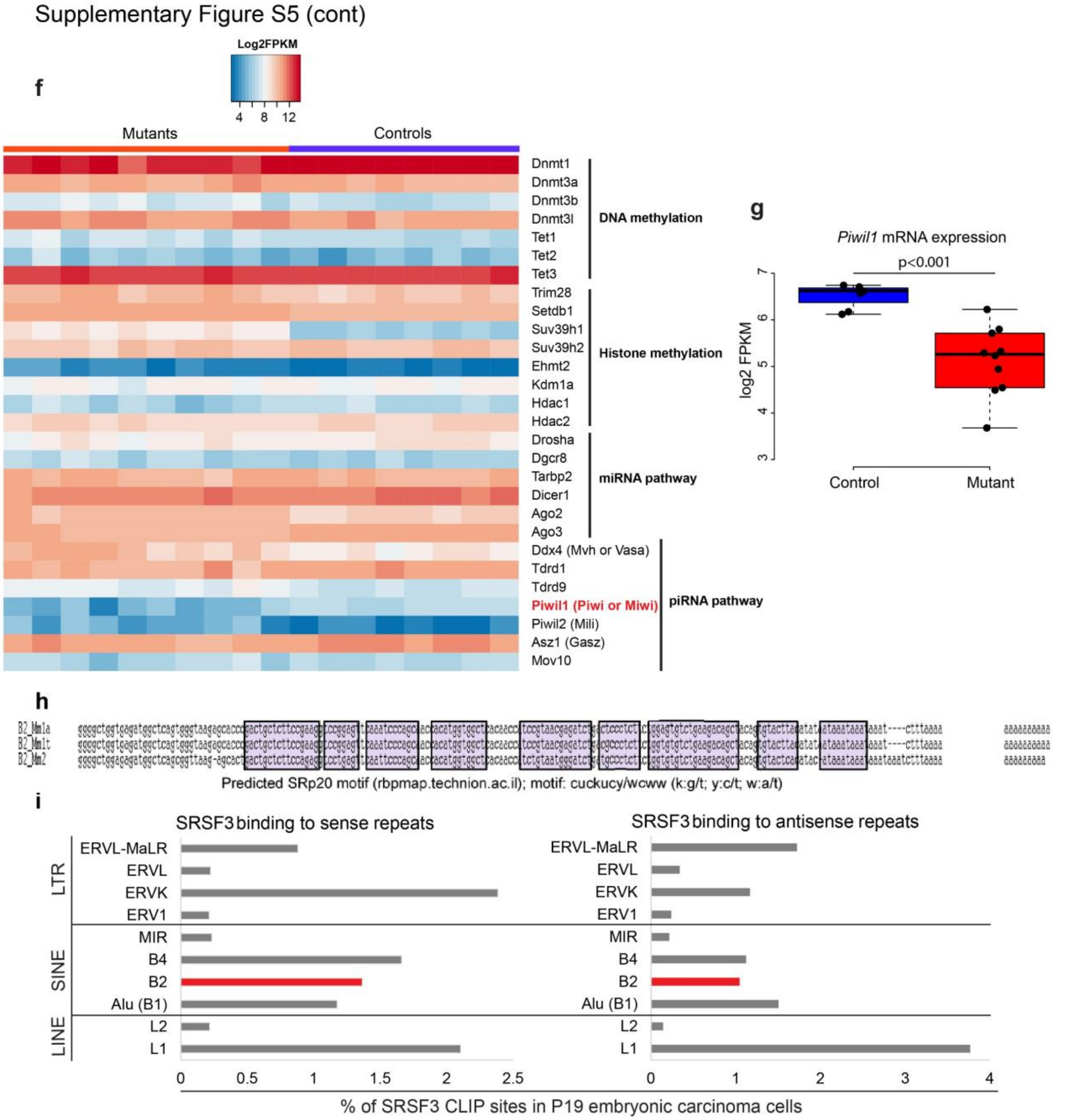

Supplementary Figure S5: Upregulated expression of B2 SINE in *Srsf3*-knockout oocytes

a. Boxplot showing the log2 fold change (mutant/control) using all reads that map to the gene
b. Boxplot showing the log2 fold change (mutant/control) using reads that map to the genes and that do not map to any SINE element.
c. Venn diagram showing the overlap of upregulated genes and upregulated SINE elements, significance was tested using Fisher's test.
d. Q-PCR result shows expression of *Srsf3* mRNA and B2 SINE RNA in single control and mutant oocytes
e. Q-PCR result shows expression of *Srsf3* mRNA and B2 SINE RNA in single control and mutant 16-cell embryos

f. A heatmap shows mRNA expression of key TE repressors in control and mutant oocytes. The colour indicates the expression level
g. A box plot shows the expression of *Piwil1* mRNA in control and mutant oocytes. Each black spot represents one sample
h. Alignment of the consensus sequence form three enriched B2 SINE classes. The boxes highlight predicted binding sites of SRSF3
i. Percentage of SRSF3 CLIP sites in repeat elements in P19 embryonic carcinoma cells

Supplementary Figure S6

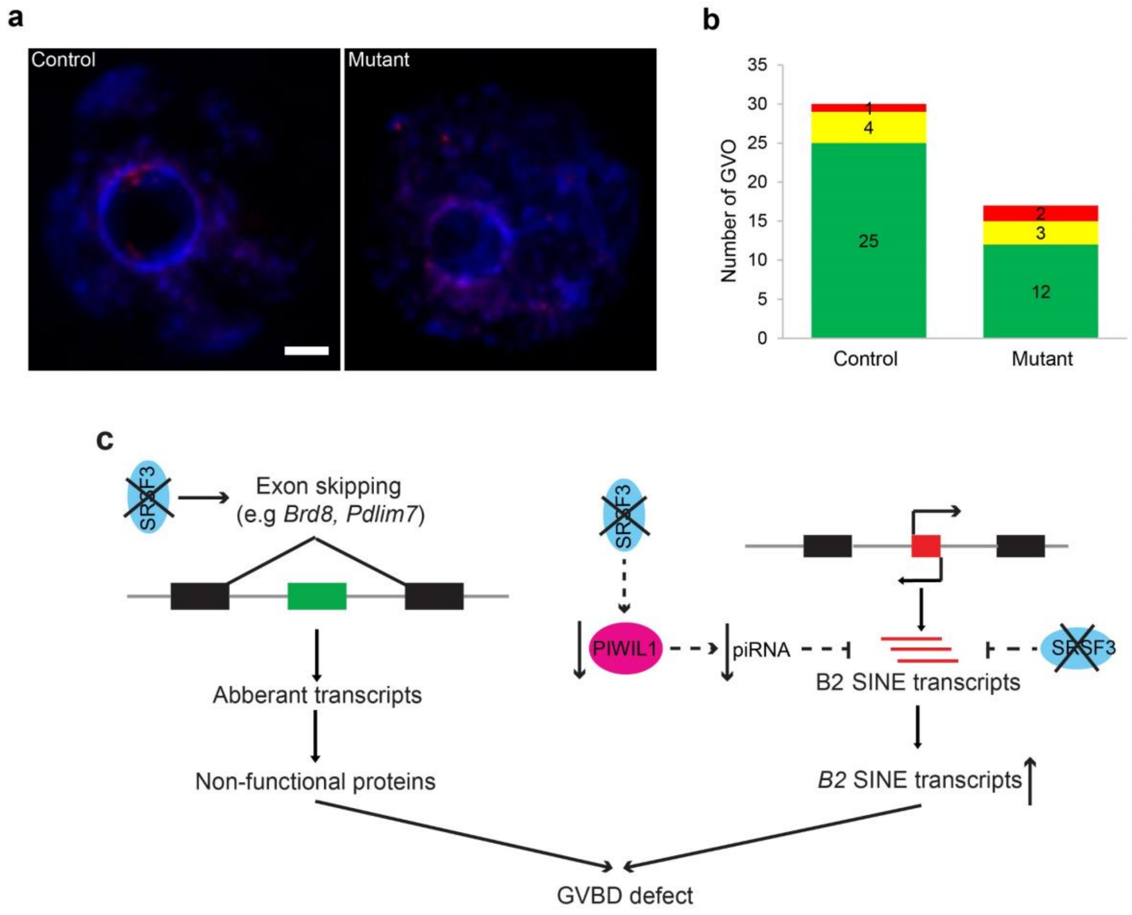

Supplementary Figure S6: No significant change in γH2AX foci in control and mutant oocytes

a. Immunostaining of γH2AX foci in control and mutant oocytes. γH2AX foci in red, DAPI in blue. Scale bar: 50 µm
b. A bar graph shows the number of oocytes with various number of nuclear γH2AX foci
c. A proposed model demonstrating that misregulated Brd8- and Pdlim7-alternative splicing and de-repression of B2 SINE contribute to GVBD defect in Srsf3-knockout oocytes. Black and green boxes represent exons, red box is alternative spliced exon, red box is B2 SINE sequence. Dashed arrows indicate hypothetical model based on bioinformatics analysis and required further future studies.

Supplementary Movie S1: A representative movie showing normal meiosis in control oocyte
Supplementary Movie S2: A representative movie showing no GVBD in mutant oocyte_1
Supplementary Movie S3: A representative movie showing delayed GVBD in mutant oocyte_2
Supplementary Table S1: Sequence of primers for RT-PCR and Q-PCR, antisense oligonucleotides for exon skipping and gapmers against B2 SINE sequence
Supplementary Table S2: List of genes with differential gene expression
Supplementary Table S3: Exon skipping events in mutant oocytes from junction reads
Supplementary Table S4: Upregulated B2 SINE transposable elements in mutant oocytes
